# Noninvasive Assessment of Portal Hypertension in Advanced Chronic Liver Disease: An Update

**DOI:** 10.1155/2018/4202091

**Published:** 2018-06-07

**Authors:** Federico Ravaioli, Marco Montagnani, Andrea Lisotti, Davide Festi, Giuseppe Mazzella, Francesco Azzaroli

**Affiliations:** ^1^Department of Medical and Surgical Sciences (DIMEC), University of Bologna, Bologna, Italy; ^2^Gastroenterology Unit, Ospedale S. Maria della Scaletta, Imola, Italy

## Abstract

The assessment of portal hypertension is a relevant step in the evaluation of newly diagnosed advanced chronic liver disease (ACLD). The current gold standard includes the invasive evaluation of hepatic venous pressure gradient (HVPG) and endoscopy. However, noninvasive or minimally invasive techniques to assess portal hypertension have been proposed and well established. In the present manuscript, we review clinical studies on the use of noninvasive or minimally invasive techniques to assess portal hypertension in ACLD patients.

## 1. Introduction

Portal hypertension (PH) is defined as an increased hepatic venous pressure gradient (HVPG) and represents a common complication of liver cirrhosis. It develops whenever resistance to portal blood flow increases because of hepatic (i.e., liver diseases), prehepatic (i.e., schistosomiasis), or posthepatic causes (i.e., Budd-Chiari syndrome). In western countries, liver cirrhosis is the most frequent cause of portal hypertension. Portal hypertension is initially asymptomatic in the vast majority of patients (around 80–90%), but when complications develop it may lead to variceal bleeding, ascites, spontaneous bacterial peritonitis, and hepatorenal syndrome among other clinical manifestations.

A review of the pathophysiology and natural history of portal hypertension is beyond the scopes of the present manuscript that focuses on noninvasive assessment of the portal pressure gradient (HVPG). However, reminding that variceal hemorrhage, ascites, spontaneous bacterial peritonitis, and hepatorenal syndrome are among the possible complications of PH is enough to underline the clinical relevance of this syndrome. Consequently, the assessment of clinically significant portal hypertension (CSPH) in cirrhotic patients is of utmost importance. Until today, the gold standard for the evaluation of HVPG is represented by transvenous catheterization of the hepatic vein.

The evaluation of portal hypertension includes, as previously suggested [1], the assessment of the pathogenic factors and of the clinical complications of portal hypertension. Among the various possible evaluations, in clinical practice, the assessment of the presence of varices and of the extent of fibrosis in the liver and spleen are certainly the most important. Recently, the Baveno VI consensus workshop [2] highlighted the diagnostic role of noninvasive techniques (NITs) such as liver stiffness measurement (LSM) in defining the presence of CSPH and EV and proposed the new term “compensated advanced chronic liver disease (cACLD)” to better define patients who present severe fibrosis and initial cirrhosis. This review will focus on these topics with a special attention to NITs.

### 1.1. Hepatic Venous Pressure Gradient (HVPG)

HVPG represents the current gold standard of HVPG evaluation and is obviously an invasive technique requiring venous catheterization. HVPG is usually within the 1–5 mmHg range and becomes clinically significant when it reaches values of 10 mmHg or above [1, 3–5]. The technique is considered safe with no fatalities reported in experienced centers and with a rate of complications of less than 1% of cases mainly represented by transient cardiac arrhythmias and local injury at the venous puncture site. It is also a technique with very few relative contraindications represented by allergy to iodinated contrast and insufficient coagulation parameters (platelets < 20,000; PT < 30%) [1, 3]. Despite this, and besides being invasive, the procedure is costly, requires expertise, and is not widely available. Therefore, noninvasive and reproducible techniques capable of substituting HVPG would be very useful in clinical practice.

HVPG is one of the best prognostic indicators so far in patients with liver cirrhosis. Several studies have highlighted the value of this technique in predicting the clinical history or the appearance of events in cirrhotic patients. The first important threshold is 10 mmHg, which defined as a cutoff of CSPH, beyond which the development of ascites, varices, and hepatorenal syndrome may be observed [6–8]. Furthermore, patients with an HVPG > 10 mmHg are at increased risk of developing hepatocellular carcinoma [9] and decompensation after hepatic resection [10]. On the other hand, an HVPG below 10 mmHg is associated with a high probability (approximately 90%) of remaining compensated over a period of 4 years [8].

When HVPG rises above 12 mmHg, the patient is at risk of variceal bleeding and ascitic decompensation [7, 11, 12]. Another important threshold is 16 mmHg that is associated with decompensation and mortality [13, 14]. Other thresholds associated with failure to control bleeding varices (20 mmHg), mortality from acute alcoholic hepatitis or alcoholic cirrhosis (22 mmHg), or spontaneous bacterial peritonitis (30 mmHg) have been identified in decompensated cirrhosis [15–18].

Apart from the abovementioned thresholds in cirrhotic patients, HVPG measurement is of relevance in evaluating the response to treatment with beta-blockers and after transjugular intrahepatic portosystemic shunt (TIPS) placement [19, 20]. In fact, a reduction of HVPG below 12 mmHg or of 20% from the baseline value equals to a reduction in the risk of development of complications and to an improved survival [21]. Failure to obtain an HVPG below 12 mmHg after TIPS placement corresponds to a dysfunction of the TIPS, and a revision of the stent is indicated [20, 22, 23]. Moreover, several studies have demonstrated a significant reduction in HVPG after achieving sustained virological response (SVR), both after an interferon-based regimen [24–26] and a DAA-based regimen [27–31], though, at the moment it is unknown which threshold can be considered as a point of “no return.”

### 1.2. Endoscopy

Upper GI endoscopy (EGD) remains the gold standard for the evaluation of the presence of esophageal and gastric varices (GEV) [32, 33]. It allows the assessment of a number of characteristics (variceal size, presence of red signs or spots, and site) that associate with the specific risk of bleeding resulting from the combination of these endoscopic characteristics, in particular to define patients with high risk varices (HRV) [34–36]. Furthermore, EGD allows the evaluation of other findings such as portal hypertensive gastropathy that may benefit from beta blockers and gastric antral vascular ectasia (GAVE) that is not specific of cirrhosis. Recommendations on the use of EGD for the detection of HRV in all cirrhotic patients have been issued since the first Baveno consensus workshop in 1992 [5]. Until the last Baveno consensus, all patients with a new diagnosis of cirrhosis must be referred to endoscopic screening to exclude the presence of GEV and in particular HRV [2]. However, a large proportion of cirrhotic patients do not present HRV, thus making endoscopy a redundant test, that is, on the other hand, associated with significant costs and patient discomfort [6]. Accordingly, in the last decade increased attention has been dedicated to identify sufficiently accurate NITs able to rule in and rule out patients who present CSPH and HRV and thus to reduce or avoid the use of invasive methods such as HVPG measurement and EGD [7, 8]. Accordingly, the so-called Baveno VI criteria stated that patients with LSM < 20 kPa (assessed by transient elastography (TE)) and with normal platelet count (PLT > 150,000/m^3^) can be considered very unlikely to have HVR (based on a reasonable risk of 5% of missed varices requiring treatment). These criteria can also be applied for longitudinal follow-up, prompting screening endoscopy if LSM increases or PLT decreases [2]. Several papers with the aim at validating the Baveno VI criteria have been published concluding that the above criteria can be safely used in clinical practice allowing to spare around 45–55% of unnecessary EGD [37, 38]. In the past year, in order to further improve the rate of spared EGD, different authors have proposed new criteria combining Baveno VI criteria with other NITs [39, 40].

### 1.3. Videocapsule

Different types of video capsules have been developed to overcome the invasiveness of classic upper GI endoscopy. However, this procedure is very expensive and the assessment of varices with this device is difficult, not comparable to the classic esophagogastroduodenoscopy and not accurate for the evaluation of gastric varices [41–45]. Therefore, this technique is not commonly used in current clinical practice to evaluate esophageal varices. Recently, Calès et al. developed an algorithm called VariScreen (a sequential combination of esophageal capsule endoscopy (ECE) with the patented CirrhoMeter test) which safely spared the missed HRV rate by 87% [46]. However, videocapsule endoscopy is not widely available and is much more expensive than conventional EGD.

## 2. Noninvasive Tests (NITs)

### 2.1. Serum Biomarkers

Many attempts have been made to detect and quantify liver fibrosis using serum biomarkers, and a series of models for fibrosis detection have been proposed. There are two main groups of tests, namely indirect and direct biomarker tests. Indirect biomarker tests are based on several serum and blood parameters that reflect liver function and progression of fibrosis to cirrhosis. Direct biomarkers are based on the measurement of factors involved in extracellular matrix turnover, which are increased in the course of liver fibrogenesis.

While serum biomarkers have been well validated for the evaluation of fibrosis in chronic viral hepatitis [47], their correlation with portal hypertension is not optimal.

Previous attempts to correlate portal hypertension or the risk of variceal bleeding with the serum levels of direct biomarkers such as laminin, type III procollagen, and hyaluronic acid did not provide affordable results [48–50]. More recently, promising results have been obtained by the measurement of the serum levels of degraded extracellular matrix (ECM) products [51].

Concerning indirect biomarkers, the performance of the majority of these scores are well investigated and validated for the diagnosis of cirrhosis rather than for the assessment of portal hypertension. Among indirect biomarkers, platelet count is probably the routinely used test able to identify patients with portal hypertension in cACLD [52].

A recent study compared the predictive value for portal hypertension of transient elastography (TE) and different indirect biomarker test panels, both as individual tests and in combination [53]. In this study, TE was compared with the AST-Platelet Ratio Index (APRI) [54], Fibroindex (a test based on platelet count, AST, and GGT) [55], and Fibrosis-4 (FIB4) (a panel based on age, AST, platelet count, and ALT) [56], both individually and in combinations of two or three of them. When individual tests were compared, TE had the best performance in terms of sensitivity (83.87%), specificity (72.53%), and accuracy (77.1%), while the association of TE associated with FIB4 had the best specificity (74.73%) and accuracy (78.8%) when a combination of 2-3 tests were considered. These results do not seem to be better than the performance of a score combining platelet count and total bilirubin reaching an 88% sensitivity and 86% specificity for the diagnosis of CSPH [57]. Similar efforts have been made by other authors who identified a good performance (AUROC > 0.70) of APRI, FIB4, and LOK score to predict CSPH [58].

Some of these serum biomarkers, alone [59, 60] or in combination with ultrasonographic parameters [61–63], have been proposed for the detection of esophageal varices. The most promising seems to be a simple score derived from the combination of acoustic radiation force impulse (ARFI) velocity and spleen diameter/platelet count [63]. This score has been developed in a training set of compensated cirrhotic patients and then validated in an external set of similar patients from a different hospital. The proposed score reached a negative predictive value of 98.3% and a positive predictive value of 100% in the validation set of patients for the prediction of the presence of high-risk esophageal varices in patients with compensated cirrhosis [63].

### 2.2. Ultrasonography

Ultrasonography (US) is a mainstay in the assessment of patients with chronic liver disease; a noninvasive, widely available, and inexpensive technique that allows the evaluation of liver morphology as well as of functional parameters with Doppler US [64–66].

Gray scale ultrasonography allows the evaluation of various elements including the liver size and its surface, the coarseness of the parenchyma, portal vein dilatation (diameter > 13 mm) and thrombosis, and the presence of signs of portal hypertension. The main signs (pathognomonic) of portal hypertension are the presence of portosystemic collaterals (flow in paraumbilical vein and development of splenorenal collaterals) and the reversal of portal vein flow. Doppler US can evaluate several parameters related with blood hemodynamics, such as portal vein velocity, congestion index, pulsatility index, and hepatic vein Doppler US pattern [64, 65, 67, 68]. However, none of these parameters allowed the grading of portal hypertension. It is clear that the findings of collaterals or of ascites associate with severe PH and a worse prognosis, however, the abovementioned US parameters show a poor correlation with HVPG and cannot, at the present time, substitute HVPG measurement [13]. Furthermore, US is operator dependent and Doppler measurement may be influenced by a number of factors such as respiration, timing of meals, steatosis, collaterals, inflammation, and equipment [69–72].

With the availability of contrast agents, other US evaluations have become possible. The measurement of hepatic vein transit times evaluated with contrast enhanced ultrasound (CEUS) have been suggested to be correlated with PH [73–76]. Of particular interest, in a study on cirrhotic compensated patients, the authors have shown a good correlation of the hepatic vein arrival time (<14 seconds) at CEUS with HVPG [77]; the AUROC for the diagnosis of clinically significant portal hypertension was 0.973 with a positive predictive value of 90% and a negative predictive value of 87–89%. Furthermore, this data is associated with the presence of large esophageal varices. Despite the promise of such good performance, the evaluation of hepatic vein arrival time has not cleared the way to routine clinical use. An explanation may come from a failure rate of about 11.5% and the variations due to the kind of software, equipment, and contrast agent used.

Another proposed parameter is the regional hepatic perfusion described by Berzigotti et al. [78] that was increased in cirrhotic patients; however, its correlation with HVPG was only weak. Conventional and Doppler US are useful in the diagnosis and follow-up of patients with liver disease, however, US signs and parameters of portal hypertension do not satisfactorily correlate with HVPG and cannot be used in place of HVPG.

### 2.3. Ultrasound Elastography Techniques

More attractive is the measurement of tissue elastography which may be accomplished by transient elastography [79–81] (TE, FibroScan®, Echosens, Paris, France) or different shear-wave-based techniques, such as point-shear wave elastography [82] (p-SWE) and two-dimensional shear wave elastography [83, 84] (2D-SWE) that have been developed and incorporated in ultrasound equipment [3, 84]. Both p-SWE and 2D-SWE are based on acoustic radiation force impulse imaging (ARFI).

Elastography techniques are commonly used for the evaluation of liver fibrosis and in the evaluation of portal hypertension [47]. A meta-analysis, performed on 18 studies, showed that liver stiffness measurement (LSM) has high accuracy (90% sensitivity, 79% specificity, and an AUC of 0.93) for the detection of CSPH [85].

Recently, the Baveno VI Consensus Conference recommended LSM values of 20–25 kPa as an accurate cutoff to identify patients with CSPH [2]; in fact, patients with LSM values > 10 kPa at TE were considered suggestive of cACLD and values of LSM ≥ 21 kPa were defined to rule in CSPH [86–88]. Despite the good results described in different studies, the main drawbacks of liver TE are the low accuracy in obese patients (an extra large XL probe is now available) and the overestimation of liver stiffness in patients with elevated ALT serum values [47].

Several studies have shown that the accuracy of p-SWE for the evaluation of liver fibrosis is comparable to that of TE [80, 89–93]. Similar to TE, p-SWE has been studied as a noninvasive tool to evaluate PH. However, studies published until now have reported conflicting results about the correlation of p-SWE and HVPG [94–96].

As expected, 2D-SWE performed as well as TE in assessing liver fibrosis with a higher accuracy in the diagnosis of mild and severe fibrosis and with a greater applicability [83, 97–99]. More recently, studies from different groups have reported a moderate or good correlation of 2D-SWE with HVPG suggesting that it might be a useful tool in the assessment of PH [100–104].

In conclusion, it is important to remember that it could be difficult to compare, mainly in terms of thresholds, the results obtained with different elastographic techniques, as was recently documented for LSM [105].

### 2.4. Spleen Stiffness Measurement

Together with significant distortions in liver and vascular architecture, alterations in spleen size and morphology are usually observed in patients with liver cirrhosis complicated by PH. It is well known that splenomegaly is a common finding in patients with cirrhosis; however, studies demonstrating a robust linear correlation between spleen size (diameter) and portal pressure are lacking [106]. Increased congestion of the splenic venous system leads to increased splenic red pulp volume and changes in histology, such as histiocyte hyperplasia, lengthening of arterial terminals, increased white pulp volume, and finally trabecular fibrosis [107, 108]. Based on these findings and the presumed consequent alteration in parenchymal strain, in the last years various groups focused their attention on the evaluation of spleen stiffness measurement (SSM) and its correlation with PH. Colecchia et al. first demonstrated a clear and reproducible correlation between SSM by TE and the presence and degree of PH assessed by HVPG [109]. With a cutoff value of <40 kPa, the authors were able to rule out the presence of CSPH with a sensitivity of 98.5%; with a cutoff value of ≥52.8 kPa, they were able to rule in the presence of CSPH with a specificity of 97.1%. Moreover, similar cutoff values have been identified to rule out and rule in the presence of EV (<41.3 kPa; sensitivity 98.1% and ≥55 kPa; specificity 95.7%, resp.) [109]. Later, many other authors found similar results also with p-SWE and 2D-SWE [94, 95, 101, 110–114]. In this setting, a recent meta-analysis of studies comparing SSM with endoscopy found suboptimal pooled diagnostic accuracy for the diagnosis of EV (pooled sensitivity 88%; pooled specificity 78%) concluding that SSM is superior to LSM (pooled sensitivity 83%; pooled specificity 66%) for predicting EV in chronic liver disease [115]. Despite these well-defined evidence, SSM has not routinely been used yet due to its technical limitation, that is, low applicability in normal-sized spleen and ceiling effect at 75 kPa impairing risk stratification of patients [66]. Technical implementations are looming: the development of a dedicated device for SSM able to detect stiffness greater than 75 kPa will be released soon [116].

Recent studies showed that SSM also has a prognostic value for decompensation events in patients with cACLD [117, 118]. Colecchia et al., followed-up for a mean of 30 months a cohort of 92 patients with HCV-related cirrhosis and found that MELD values and SSM were independently correlated to the risk of decompensation. The authors identified a SSM cutoff value of 54 kPa able to identify patients with a low risk of decompensation (negative predictive value 0.975) and proposed the use of SSM as a noninvasive prognostic factor in patients with compensated HCV cirrhosis [117]. Up to now, the MELD model score and the Child Pugh score are mainly used in clinical practice as independent predictive scores of mortality. Recently, Takuma et al. [95] assessed SSM by SWE to predict mortality as a primary end point; the authors found that SSM had the best discriminative value among all other clinical variables; each SS unit (m/s) of increase by p-SWE was associated with a 14.5-fold increase in the risk of death, and the cutoff of 3.43 m/s had a 75.8% accuracy in predicting mortality after a median follow-up of 44.6 months. Recently, a small but intriguing study demonstrated a rapid reduction of SS in patients before and after liver transplantation (LT); the authors investigated 21 patients awaiting for LT and 11 patients after LT. They hypothesized that the resolution of PH consequent to LT could reflect into significant changes in SSM. Patients awaiting for LT showed a SS measurement significantly higher compared to posttransplant patients (75 versus 28.4 kPa; *P* < 0.0001). The authors demonstrated that SS noninvasively reflects changes in PH after LT, even in the early phases post-LT [119]. In a similar setting, SSM was demonstrated to be a valid tool for the assessment of early changes of portal hemodynamics: two different studies evaluate changes of SSM after transjugular intrahepatic portosystemic shunt (TIPS) creation. The authors found nonunivocal results: while Gao et al. found a significant reduction of SSM in 10 patients after TIPS placement (3.65 m/s versus 3.27 m/s; *P* < 0.001), Novelli et al. found an increase in SS in 42% (8 out of 19) of patients and a reduction in the remaining 58%. Concurrent coil embolization of portal collateral was correlated to the increase of SSM, but the small study population does not allow performing detailed correlation (resulting in an underpowered multivariate analysis) [120, 121].

Finally, the application of NITs for the diagnosis and grading of PH and its complication is even more important in a pediatric population, in which the performance of invasive procedures (i.e., upper endoscopy) have to be performed under deep sedation, with a significant increase in cost and related morbidity. In this field, some authors evaluated the application of SSM as a reliable tool to spare unnecessary maneuvers. Goldschmidt et al. performed SSM in 99 children with different degrees of chronic liver disease; in the pediatric population, SS measurement using TE was feasible in 90.5% of children with splenomegaly (versus 70.2% in children without). The authors found a significant increase of SS in patients with varices (75 versus 24 kPa) and found that patients with SSM < 60 kPa had no risk of upper variceal bleeding [122].

### 2.5. Indocyanine Green Clearance

Several serum markers of the liver function test have been developed and evaluated in order to obtain detailed and reliable information on functional reserve. Indeed, in this field, the routine application of liver function tests (i.e., serum albumin, bilirubin, INR, etc.) or even composite score (i.e., MELD, Na-MELD, etc.) is usually quite insensitive and nonspecific. Dye test, measurement of total serum bile acid concentration, breath tests, and metabolic clearance tests (i.e., caffeine) have been tested and demonstrated able to estimate hepatic excretory capacity. Despite known clinical potential applications, these tools are more complex to perform and to reproduce and thus rarely used in clinical practice [123, 124].

The estimation of liver function reserve by a clearance test is based on the assessment of the synthetic capacity through the administration of a known substrate with the measurement of a known product or clearance of an exogenous drug, which is mostly removed and/or metabolized by the liver. Metabolic clearance depends upon three major factors, hepatic perfusion, sinusoidal exchange, and functional hepatic mass. Thus, clearance of substrates dependent upon microsomial function (i.e., aminopyrine or caffeine) reflects functional reserve, while a substrate with high extraction (i.e., indocyanine green) depends mostly on blood flow [125, 126].

Indocyanine green (ICG) is a water-soluble organic dye that binds to albumin and alpha-1 lipoproteins; after an active uptake from hepatocytes, it is secreted unchanged into the bile. ICG is removed exclusively from the liver and does not undergo enterohepatic circulation. Finally, ICG clearance is modified by acute changes in vascular liver perfusion [127]. ICG clearance is a quantitative liver function test representing both parenchymal function and hepatic blood flow. Based on its characteristics, ICG clearance is routinely used, especially in Eastern countries, for the assessment of liver function in patients undergoing hepatic surgery (hepatocellular carcinoma or biliary cancer resection) [128, 129].

Our group evaluated the ability of ICG clearance to evaluate the presence and degree of PH and its complications (EV) among patients with compensated cirrhosis. We hypothesized that, among patients with a well-preserved liver function, the ICG-retention test will directly reflect liver blood flow and thus, indirectly, the presence of PH. In a homogeneous group of well-compensated (child A) cirrhotic patients, we observed a good direct correlation between the ICG-retention test at 15 minutes (namely ICG-r15) and HVPG measurement [130]. Among noninvasive serum markers tested, ICG-r15 showed the best diagnostic performance for the assessment of portal hypertension, CSPH, and SPH; indeed, according to ROC curve analyses, we identified two cutoff values (<6.7% and <6.9%) able to rule out the presence of CSPH and SPH with a very good sensitivity (95.9% and 96.6%, resp.). These preliminary data have been further validated by an independent Danish group [131].

Moreover, ICG-r15 seems to be a valid noninvasive tool for ruling out the presence of EV; we identified two cutoff points: ICG-r15 < 10% able to rule out the presence of EV (sensitivity 97.8%, NPV 96.3%, and negative LR 0.042) and ICG-r15 > 22.9% to rule in the presence of EV (specificity 90.0%, PPV 83.8%, and positive LR 5.43). We also observed that 45 out of 46 patients with EV were correctly identified by the proposed cutoff, which is able to exclude the presence of large EV with a sensitivity of 100% and a negative likelihood ratio of 0.0 [130].

It is well known that quantitative liver function tests are able to detect early changes in liver blood flow and function with good reproducibility; their ability to show functional impairment reflects on the usefulness as prognostic factors for short-term clinical outcomes [132]. To date, a relevant study confirmed that the incorporation of ICG clearance into MELD (MELD-ICG) increases accuracy on the prediction of 1-year survival in patients with intermediate-advanced liver cirrhosis (area under ROC curve for MELD: 0.58–0.71 versus MELD-ICG: 0.65–0.73). The authors hypothesized that the integrated information on blood flow to liver function leads to the increased accuracy as a prognostic factor [133].

Liver disease progression is characterized by hemodynamic alterations occurring together with liver functional impairment; we recently evaluated if ICG-r15 could be a long-term prognostic factor in patients with compensated disease because of its demonstrated ability to provide information that integrates the assessment of liver blood flow and hepatic functional reserve.

We longitudinally observed 134 patients for a mean of 29 months. Our preliminary observation showed that ICG-r15 (OR 1.068; 95% CI 1.038–1.098), HVPG measurement (OR 1.100; 95% CI 1.017–1.190), and presence of EV (OR 2.544; 95% CI 1.141–5.673) are independently correlated with the development of decompensation. Kaplan-Meyer curves confirmed ICG-r15 ≥ 22.9% (HR 5.491; 95% CI 2.681–11.245), HVPG ≥ 12 mmHg (HR 2.686; 95% CI 1.456–4.954), and presence of OV (HR 5.050; 95% CI 2.184–7.511) as risk factors for decompensation [134].

### 2.6. Magnetic Resonance (MR) and Computed Tomography (CT)

In clinical practice, at least in Europe, MR and CT are not frequently used just to evaluate PH. The reason relies on costs and ease of access, particularly for MRI. In fact, in the United States, CT scan is cost-effective for the diagnosis of gastroesophageal varices [135], but the technique is less costly than in Europe, where endoscopy is usually cheaper. About CT, scan irradiation represents another factor weighing on its use.

This radiologic technique can give an accurate representation of the morphology of the portal venous system and on the presence and extent of thrombosis as well as on the presence of collaterals. This accurate imaging may be particularly useful before TIPS placement in patients with posthepatic portal hypertension such as in the Budd-Chiari syndrome. With regard to varix detection, the performance of CT and MRI are good for large varices, but lower for small varices [3, 136–138].

Another application of MR is the elastography of the liver (MRE) [139]. Recent meta-analyses have reported good results for MRE in the evaluation of fibrosis [140, 141]. Differently from US-based methods, MRE allows the evaluation of the entire liver and is not limited by body habitus or meteorism [142, 143]. However, long-term studies are not available yet and, as abovementioned, its use is limited by costs.

Promising results have been reported also for the evaluation of spleen stiffness elastography using a MRE protocol comparing healthy controls and patients with chronic liver disease (CLD) [144]. MRE was successfully performed in all patients, and the authors found a significant SSM difference among healthy volunteers and patients with liver fibrosis (3.6 ± 0.3 versus 5.6 ± 5.0 kPa; *P* < 0.001). Interestingly, the authors identified a SS cutoff value of ≥10.5 kPa able to identify the presence of esophageal varices in patients with compensated cirrhosis with a specificity of 100% [144]. These preliminary data have been recently confirmed by other groups; in particular, Shin et al. showed that MRE was highly reproducible and could be integrated with double contrast-enhanced MRI to increase the sensitivity and overall accuracy for the diagnosis of EV [145]. Multiparametric liver and spleen MRE have been correlated with HVPG measurement and endoscopy [146]. The authors calculated three parameters, namely storage, loss, and shear moduli. Among them, spleen loss modulus appears to be the best parameter for identifying patients with severe portal hypertension (AUC 0.81) or high-risk varices (AUC 0.93).

## 3. Conclusions

The armamentarium available to hepatologists for the assessment of PH has increased in recent years with the advent of several NITs beside the classical and invasive measurement performed by HVPG and endoscopy. The availability of each technique is not universal since some of them are quite expensive and require specific expertise. In this scenario, the availability of easily reproducible techniques with acceptable costs is certainly welcome.

While HVPG measurement and endoscopy remain the gold standard for the evaluation of PH and varices, NITs will likely optimize their use. We think that in a patient with a newly diagnosed cACLD, a screening with NITs is preferable in order to define the best timing to perform endoscopy or other invasive techniques unless we are facing a decompensated patient. The real point is when to perform these procedures in patients with an initial-stage ACLD that are unlikely to have already developed CSPH.

With regard to the techniques described in the present paper, we believe they have the potential to partially satisfy this request either alone (i.e., indocyanine green and SSM) or in combination (i.e., Baveno VI criteria and other NITs). The choice of the technique, excluding significant differences in accuracy that may arise as our knowledge expands, will likely be dependent on local availability and expertise. For example, since ultrasonography is already current practice for liver patients, it is likely that the implementation of elastographic techniques on already available ultrasound machines will have a larger diffusion than TE. Furthermore, it allows performing ultrasound and elastometric examination at the same time and has a higher success rate.

In times during which we have to face with monetary budgets, choosing the right time to perform an appropriate procedure is advisable in order not to waste money.
